# Supplementary figures and images for: Spatial modeling could not differentiate early SARS-CoV-2 cases from the distribution of humans on the basis of climate in the United States
Source: PeerJ. 2020 Oct 26;8:e10140. doi: 10.7717/peerj.10140 (PMC7594635; doi:10.7717/peerj.10140)

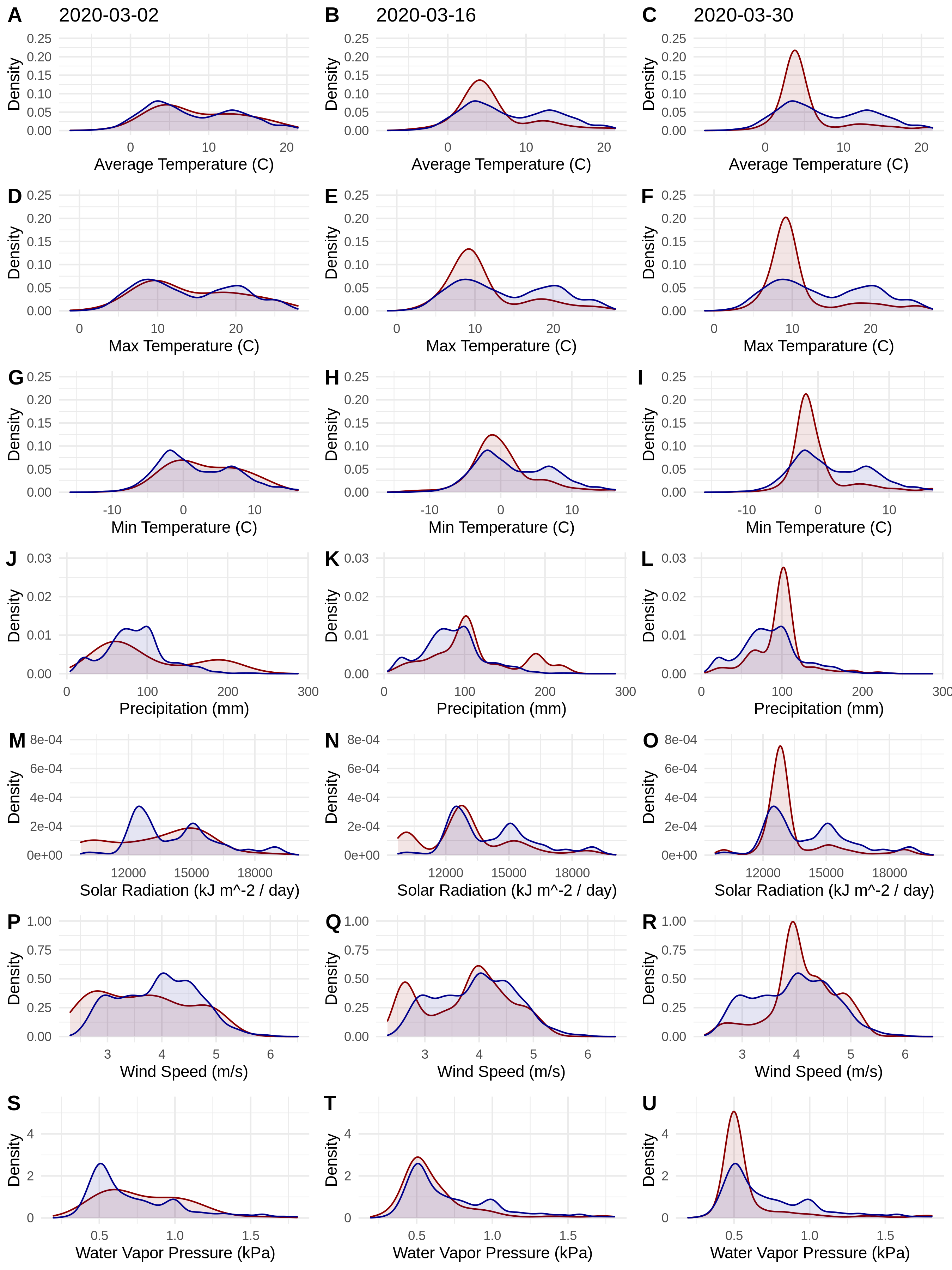

Supplement: Supplemental Information 1 — Probability densities of SARS-CoV-2 coronavirus cases (using raw data; curves in red) compared to the probability densities of human populations (curves in blue) in each US county for each of seven climate variables. Probability density curves are standardized to an area of one. [file peerj-08-10140-s001.png]

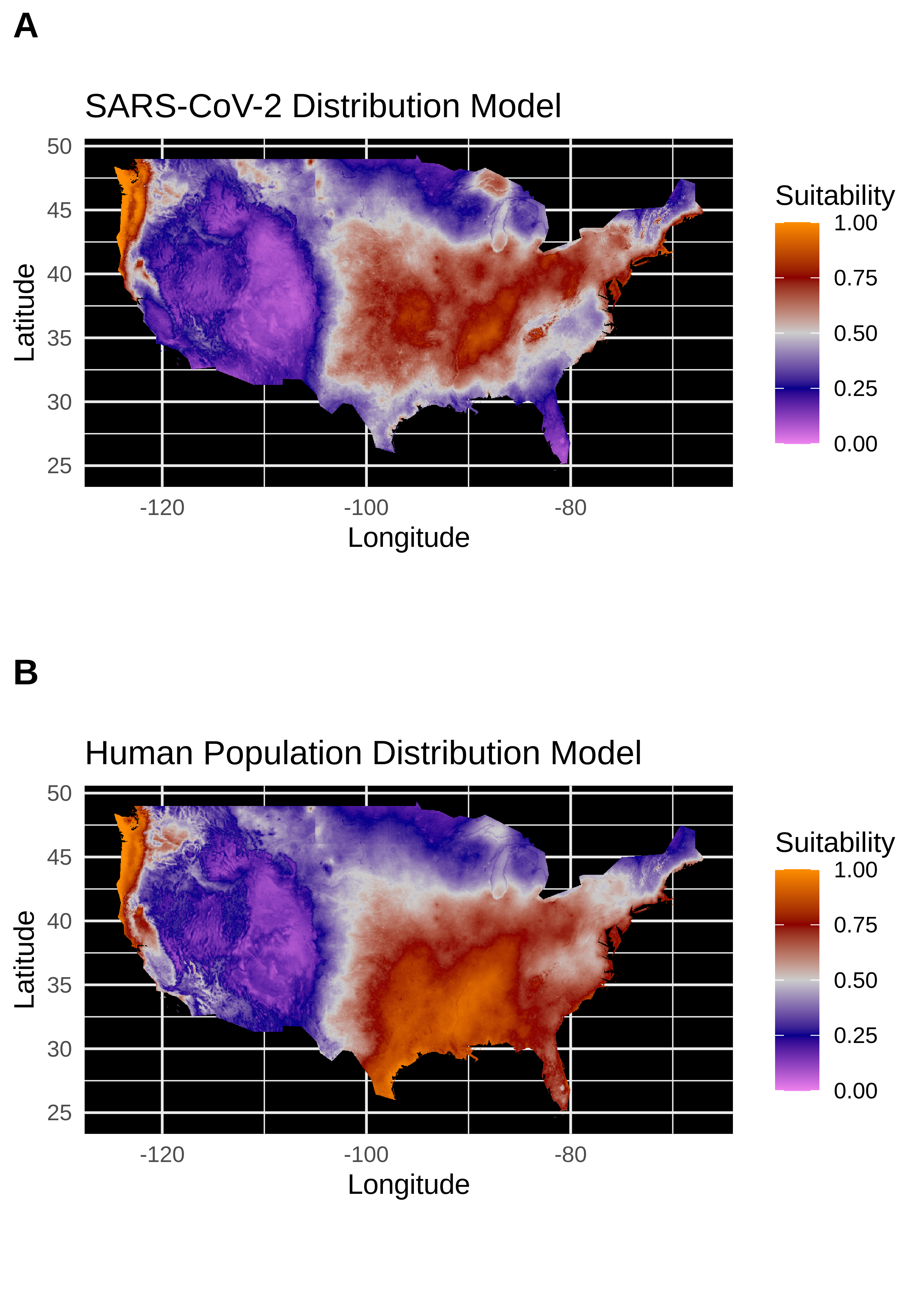

Supplement: Supplemental Information 2 — (A) Species distribution model of the SARS-CoV-2 coronavirus (using raw data) for March 30, 2020. (B) Human population distribution model for the US from 2010. [file peerj-08-10140-s002.png]

## Overlap

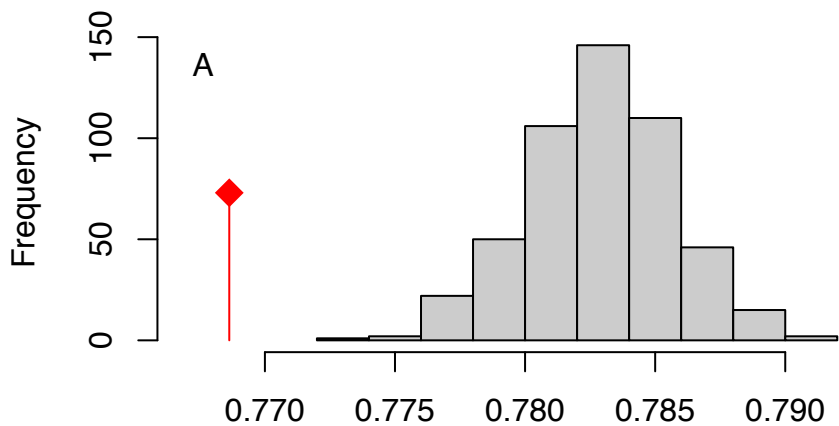

D  
 $p\text{-value} = 1$

## Similarity

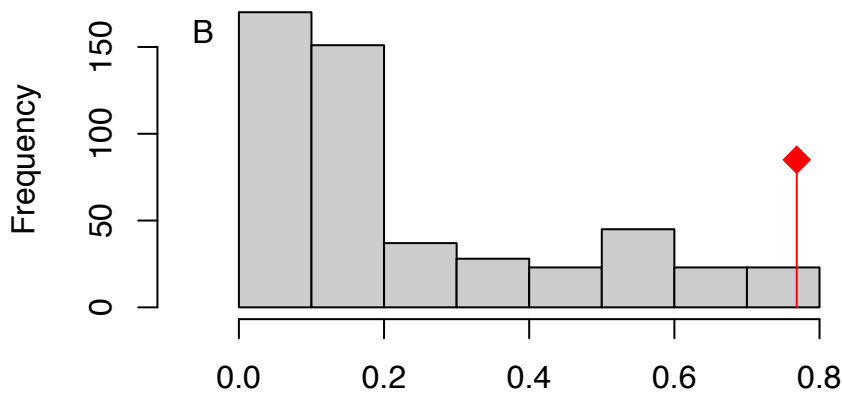

D  
 $p\text{-value} = 0.01996$

Supplement: Supplemental Information 3 — (A) Niche Overlap and (B) similarity tests for Maxent species distribution models built with SARS-CoV-2 coronavirus case data compared to one built with human population density as occurrence data; actual model overlap indicated by a red marker in both plots. Significant p-values correspond to greater niche overlap or similarity than expected by random models. [file peerj-08-10140-s003.pdf]
